# Assessment of Physical Activity During Radiation Therapy for Lung Cancer: Study Protocol of the APART-LUNG Study

**DOI:** 10.3390/clinpract16040080

**Published:** 2026-04-20

**Authors:** Dirk Rades, Maria Karolin Streubel, Laura Doehring, Stefan Janssen, Sabine Bohnet, Christian F. Schulz, Hanne Falk Grauslund, Charlotte Kristiansen

**Affiliations:** 1 Department of Radiation Oncology, University of Lübeck, 23562 Lubeck, Germany; stefan.janssen@uksh.de; 2 Department of Radiation Oncology, University Medical Center Schleswig-Holstein, Lübeck Campus, 23538 Lubeck, Germany; mariakarolin.streubel@uksh.de (M.K.S.); laura.doehring@uksh.de (L.D.); 3 MVZ RON Niedersachsen Strahlentherapie Gesellschaft mit beschränkter Haftung (GmbH), 30161 Hannover, Germany; 4 Department of Pulmonology, University Medical Center Schleswig-Holstein, Lübeck Campus, 23538 Lubeck, Germany; sabine.bohnet@uksh.de; 5 Department of Radiation Oncology, University Medical Center Schleswig-Holstein, Kiel Campus, 24105 Kiel, Germany; christian.schulz@uksh.de; 6 Department of Oncology, Vejle Hospital, University Hospital of Southern Denmark, 7100 Vejle, Denmark; hanne.falk.grauslund@rsyd.dk (H.F.G.); charlotte.kristiansen@rsyd.dk (C.K.)

**Keywords:** lung cancer, radiation therapy, conventional fractionation, physical activity, step counter

## Abstract

**Background/Objectives**: Radiation therapy is a common treatment modality for non-small-cell and small-cell lung cancer that can be associated with considerable side effects, mainly reactions of healthy tissues in the radiation field. Radiation therapy may lead to significant fatigue, which can potentially be mitigated by maintaining or increasing physical activity during treatment. Since achieving this goal may be a challenge for patients, they may benefit from a mobile application reminding them daily to perform a predefined number of steps. Such a reminder app will be investigated prospectively in a phase 2 trial. The current APART-LUNG study (NCT07380815) is a mandatory study for designing the prospective trial. **Methods**: The main objective of the APART-LUNG (exploratory non-interventional) study is to report patterns of physical activity during radiation therapy for lung cancer patients and generate hypotheses based on our findings. Our primary endpoint is the within-patient difference in weekly average steps per wear hour of the smartphone (week 5 minus week 1 of radiation therapy), and our secondary aim is to estimate differences in operational measures (wear time of the smartphone) between week 5 and week 1. The sample size of approximately 20 patients (full analysis set) allows us to detect a moderate-to-large standardized within-patient difference and is driven by feasibility and the intent to obtain preliminary estimates of effect size and variability. The results of the APART-LUNG study will be very important for appropriately designing a phase 2 trial.

## 1. Introduction

A considerable number of patients with lung cancer receive radiation therapy [1,2], an anticancer treatment that can lead to considerable side effects, including fatigue, which may result in a reduced intensity of physical activity [3,4,5,6,7,8,9]. Patients who experience significant fatigue may be unable to complete their entire radiation therapy course as initially planned.

The importance of exercise and physical activity for lung cancer patients has been highlighted in several review articles [10,11,12,13,14]. In a study including cancer patients, of whom 31 were treated with chemotherapy for lung cancer, exercise was shown to lead to fewer dose reductions and treatment delays [15]. Furthermore, patients’ quality of life may be improved if they are physically active [16,17,18,19,20,21,22]. In one retrospective study of lung cancer patients treated with chemotherapy, a relationship between physical activity and time to death was observed [23], supporting the importance of physical activity during anticancer treatments such as radiation therapy. Adherence to regular exercise can be a challenge for patients, particularly if side effects occur [24,25]. The best option to increase physical activity will likely be in-person kinesiology activities, such as in-person group exercise programs, physical therapy, or yoga programs. Videos, online courses, and mobile applications (reminder apps) may be less effective but could potentially be helpful. As part of the Interreg project Health Advancing Technologies for Elderly (HeAT), an app for patients’ smartphones that reminds them several times a day to walk a predefined number of steps has been created. The potential effect of this reminder app on a patient’s physical activity will be tested in a phase 2 trial. However, the step counts derived from smartphones are influenced by variability in device carrying time, and this variability introduces measurement heterogeneity that must be explicitly addressed in the statistical design and analysis. This study is a pilot investigation for a phase 2 trial evaluating changes in physical activity during radiation therapy and is a prerequisite for designing the phase 2 trial. The APART-LUNG study was approved by the German ethics committees of the University of Lübeck and the Christian Albrechts University, Kiel.

## 2. Study Design and Materials

### 2.1. Objectives and Endpoints

The primary objective of the APART-LUNG study is to estimate the within-patient changes in physical activity between week 1 and week 5 of radiation therapy, measured using smartphone-recorded step counts normalized for the device-carrying time. Week 5 of radiation therapy was chosen because we would like to receive information about the patients’ physical activity during the treatment course and since most patients treated with conventional fractionation receive at least 50 Gy administered over five weeks (five fractions of 2.0 Gy per week). To account for inter- and intra-individual variability in device usage, normalized daily step counts are calculated as the total daily steps divided by the estimated daily smartphone carrying time expressed in hours. Our main question relates to the course of the mean number of steps during the weeks of radiation therapy, namely, whether it will remain almost constant or become lower. The latter option appears more likely (hypothesis). Our secondary objectives are to examine operational measures such as wear times, to assess the feasibility of the primary endpoint, and to explore heterogeneity in the primary endpoint across relevant subgroups (considering the rather limited sample size of this study).

The APART-LUNG study is a pre-study for a prospective phase 2 trial that will investigate the value of a reminder app. This study is necessary for the optimal creation of the study protocol and calculation of the required number of study participants, and the cohort of the present study will serve as a control group for the phase 2 cohort using the new reminder app.

Our secondary objective is to estimate the differences in operational measures (wear time of the smartphone) between week 5 and week 1. In addition, the feasibility of the primary endpoint will be assessed, including the proportion of patients with both weeks evaluable and operational reasons for non-evaluable pairs. Moreover, the heterogeneity of week 5 and week 1 changes across subgroups, namely, age categories (<65 vs. ≥65 years), pre-treatment performance status, and histology (NSCLC vs. SCLC), will be explored.

### 2.2. Trial Design and Duration

The APART-LUNG study is a multicenter, single-arm study (exploratory non-interventional study) in lung cancer patients previously treated with radiation therapy, in which physical activity is assessed at week 1 and week 5 of radiation therapy using a smartphone application. Approximately 20 patients are required for the full analysis set, and recruitment will be completed within 3 months (=total running time).

### 2.3. Eligibility Criteria

Inclusion and exclusion criteria for the APART-LUNG study are given in Table 1.

### 2.4. Sample Size Calculation

The study is designed as a pilot and feasibility investigation and is not intended to provide confirmatory evidence. The sample size of approximately 20 patients is driven by feasibility and the intent to obtain preliminary estimates of effect size and variability, and formal hypothesis testing power is not targeted. Nevertheless, an illustrative sample size justification based on standardized differences is provided to support the planned enrollment of approximately 20 patients: the standardized effect size for the primary endpoint is defined as Cohen’s d for paired data, calculated as the mean within-patient difference in weekly normalized step counts divided by the standard deviation of these differences. With a sample size of 20 patients, a two-sided significance level of 5%, and 80% power, the study is capable of detecting standardized within-patient differences of approximately 0.6 to 0.65, corresponding to moderate-to-large effects. This level of sensitivity is considered appropriate for the exploratory objectives of the study. The sample size allows for a reasonably precise estimation of effect sizes and variability while remaining feasible in the context of a pilot investigation. All inferential results will be interpreted descriptively and will primarily serve to inform the design of future confirmatory trials.

## 3. Detailed Procedure

### 3.1. Overview of Examinations and Data Assessed at Baseline

An assessment of the data required for the APART-LUNG study will be performed in Germany (Hannover, Kiel, and Lübeck) and Denmark (Vejle), and an overview of assessments and examinations at each visit is given in Table 2. The following data are assessed at the time of baseline but refer to the time of radiation therapy: medical history, medication, demographics, body height and weight, Karnofsky performance score, histology, and tumor stage.

### 3.2. Examinations Related to Primary and Secondary Aims

Number of daily steps: Physical activity is documented using total daily step counts recorded by a smartphone-based application under real-life conditions, obtained for the first and fifth weeks of the patient’s radiation therapy course from the memory of the patient’s smartphone. Suitable patients treated with conventionally fractionated radiation therapy between 2023 and 2025 who own a smartphone that was used during treatment, had a step-counting application active during radiation therapy with stored historical data, and are willing and able to return to the institution are asked to visit the institution where they received their radiation therapy for lung cancer. Patients will be initially contacted by phone to check whether they have a smartphone and a step counter with a memory function and whether they are interested in potentially participating in this study. Patients will provide written informed consent when they visit the institution where they were irradiated. If the patient states that they do not require an additional reflection period, an assessment of the steps taken during the first and fifth weeks of the radiation therapy course from the memory of the patient’s smartphone will be performed by an investigator trained for the APART-LUNG study. The type of smartphone will be documented. After manual extraction of the step count for every single day from the patient’s smartphone, the data will be documented in the case report form. Subsequently, the daily steps will be added together and divided by the number of days to obtain the average number of steps.

#### Time per Day the Smartphone Was Worn Close to the Body

Patients will be asked to rate the time (average hours per day) that their smartphone was worn close to their body during the first and fifth weeks of their radiation therapy course.

### 3.3. Statistical Considerations

#### 3.3.1. Primary Aim

Our primary objective is to describe the pattern of physical activity during a course of radiation therapy, measured using total daily step counts recorded by a smartphone-based application. In addition to step counts, patients’ estimates of their mean daily wear hours in week 1 and week 5 are documented. If applicable, the wear hours may be determined from the health app on the patient’s smartphone. Week 1 is defined as days 1 to 7 following the initiation of radiation therapy, and week 5 is defined as days 29 to 35. For each patient, daily normalized step counts are calculated as described in Section 2.1. After discussion with an experienced statistician and several clinicians, a valid day is defined as a day with a minimum of eight hours of wear time, and a valid week means at least three valid days within the specified week. This threshold is not evidence-based but is rather chosen to ensure that the daily step counts reasonably reflect overall daily activity. Since no data have been found in the literature regarding this topic, the definition of a valid day may be adjusted during the course of the study to reflect a realistic scenario.

The primary endpoint is the within-patient difference in the normalized average step count per week (week 5 minus week 1). No imputation of invalid normalized average step counts per week is planned for the primary analysis, and the extent and pattern of missing data will be recorded descriptively. Given the exploratory nature of the study and the small sample size, advanced imputation methods are not planned. In addition to hypothesis testing, point estimates (e.g., mean or median change) and interval estimates (e.g., 95% confidence intervals) will be reported to quantify the magnitude and uncertainty of the observed within-patient change.

The primary estimand targets the average within-patient change in physical activity between radiotherapy weeks 1 and 5, and the population of interest comprises adult patients undergoing radiation therapy who use a smartphone-based step-counting application. Relevant intercurrent events such as incomplete smartphone carrying, temporary technical failures, changes in radiation therapy, and hospitalization may lead to missing step count data. These intercurrent events are addressed using a treatment policy strategy whereby all available valid data are included in the analysis, and days without sufficient carrying time are excluded without further adjustment. This estimand reflects the change in physical activity during radiation therapy as observed under real-world conditions of smartphone usage.

To describe the individual trajectories of the normalized average step count per week between week 1 and week 5, spaghetti plots will be created. In addition, summary statistics stratified by week will be provided for subjects with non-missing data, including the mean, standard deviation, median, first and third quartiles, minimum, and maximum. The normalized average step counts per week will be summarized using the same statistics. This analysis allows us to explore whether the normalized average step counts per week changed between week 1 and week 5. Given the modest sample size and the exploratory nature of the study, the Wilcoxon signed-rank test will be used as the primary inferential method, while a paired *t*-test may be reported as a supportive analysis if the distribution of within-patient differences is approximately normal. If diagnostic checks indicate material skewness or heteroskedasticity, a supportive analysis on the log scale will be presented as a ratio of geometric means with a 95% confidence interval, without changing the primacy of the original scale analysis.

The magnitude of the activity change will be quantified by estimating the mean within-patient difference together with the 95% confidence interval, interpreted descriptively. Standardized effect sizes for paired data (Cohen’s d) will also be reported to support interpretation and future planning.

A linear mixed-effects model with a fixed effect for time (week 1, week 5) and a random intercept for the patient to account for repeated measures will also be applied for exploratory analysis of the primary endpoint. The primary contrast estimates the adjusted mean difference between week 5 and week 1. The model reports the point estimate, a two-sided 95% confidence interval, and a descriptive *p* value. The primary analysis is on the original scale; in the case of skewness, a supportive analysis is performed on the log-transformed data, and the result is expressed as a ratio of geometric means with a 95% confidence interval. Exploratory subgroup analyses will assess whether the week 5 versus week 1 change differs across categories of relevant subgroups (for example, age category, performance status, and tumor type). The mixed-effects models are exploratory only, intended to identify potential trends for future adequately powered studies, and the present study is not intended to draw confirmatory statistical conclusions but to generate insights that may guide the design of subsequent, more robust clinical investigations. The combined approach, supplementing robust paired testing for the primary endpoint with exploratory trajectory modeling, maximizes insights from a limited sample while maintaining methodological appropriateness for a pilot study.

#### 3.3.2. Secondary Aim

Descriptive methods will be applied to analyze the secondary aims. Subgroup analyses will only be considered if the sample size of the respective categories is sufficiently “large”. In this case, a time x subgroup interaction will be added to the primary model, and subgroup-specific paired differences with 95% confidence intervals will be reported.

### 3.4. Ethical and Legal Principles

The study protocol was submitted to the responsible ethics committee, and a positive vote was communicated to the trial management. Patient information, combined with informed consent, was also submitted to the ethics committee. Before inclusion in the study, each patient will be informed about the content and procedure of the study and will receive a copy of the signed form. The investigator must also inform the patient about the right to withdraw consent to participate at any time and without giving any reasons and that the data collected as part of the study will be documented anonymously and then forwarded for scientific evaluation. The study will be performed in accordance with the principles laid out in the Declaration of Helsinki.

### 3.5. Data Protection and Data Management

Details of data protection and data management have been previously reported for other prospective trials from the Interreg project HeAT [26,27,28]. The same applies to the distribution of the results. Collection of the data will occur according to the regulations of the Data Protection Act, and patient-related data will be recorded pseudonymously, handled in accordance with general data protection regulations, and analyzed by a professional external statistician following a previously defined statistical analysis plan. Study-related documents will be stored for 10 years. Scientific publications related to the study are reviewed by the external statistician. Moreover, the acronym APART-LUNG will be used in the corresponding publications.

## 4. Expected Results and Discussion

Lung cancer patients receiving conventionally fractionated radiation therapy with total doses up to 70 Gy may develop significant side effects [24,25] and can have difficulties in maintaining their degree of physical activity, which can subsequently negatively impact livability.

One can imagine that, for patients suffering from a locally advanced malignant disease who are under strenuous treatment, it may be quite difficult to follow an exercise program and maintain their pre-treatment activity level. While these patients will likely benefit from in-person activities, an app reminding them regularly to be physically active may also be helpful. Such an app has been recently designed [27] and can be downloaded by the study participants on their smartphones. Since the required data will be obtained directly from the patients’ smartphones, no external data transfer is needed. The development of such an app is in line with current trends in radiation oncology. Apps have been created or are under development with different purposes for patients undergoing irradiation [29,30,31,32]. The recently created app will be tested in a prospective phase 2 trial. For the optimal creation of the study protocol and calculation of the required number of study participants, a pre-study is necessary that addresses patterns in the number of steps taken during radiation therapy, namely, whether it remains almost constant or decreases. The APART-LUNG was initiated to answer this question and contribute to the design of the planned phase 2 trial. Moreover, the cohort of the APART-LUNG study will serve as the control group for the subsequent prospective trial.

### Limitations of the Study

When using the findings of the APART-LUNG study for the phase 2 trial, the limitations of the current study should be kept in mind. One major limitation is the fact that the step counts examined in this study were made during the period of radiation therapy and cannot be assessed in a truly prospective manner. The fact that the study is not designed as a fully prospective observational cohort study but relies on a retrospective retrieval of step count data and a retrospective self-report of smartphone wear time introduces substantial methodological limitations. The fact that the patients estimate the average daily wear hours retrospectively bears the risks of recall bias, measurement error, and potential differential misclassification, which may significantly impact the validity of the normalized step metric. However, when designing the study, the involved experts believed that the wear time would likely not differ significantly between the two weeks of interest. Moreover, the wear time could be determined from the patients’ smartphones or smartwatches (health apps). In a prospective study, wear time could be recorded contemporaneously, the step count data could be monitored in real time, and data completeness could be verified during the course of radiation therapy. The patients included in the prospective trial should be willing to wear their smartphone close to their body, at least during the time they are awake. In a previous randomized pilot study investigating the impact of physical activity on quality of life and biomarkers in lung cancer patients, participants were required to permanently wear an accelerometer except during the time it charged [33]. Moreover, additional important data can be obtained from a prospective trial, including quality of life, fatigue, mood, hopelessness, pain, sense of well-being, appetite, leisure activities, sleeping habits, and aspects of emotional distress such as anxiety, depression, worry, fears, sadness, nervousness, and loss of interest in usual activities [34,35]. Moreover, the impact of radiation therapy on the levels of cytokines (predominantly IL-6 and IL-8), the neutrophil-to-lymphocyte ratio, and the complete blood count could be explored [36,37]. Additional potential biases include a selection bias due to the fact that the patients are required to own a smartphone and a step counter and that the patients have to still be alive and available at the time of the study. Moreover, the small sample size, while adequate for a feasibility and variability pilot, limits statistical precision and the generalizability of the results across specific subgroups (e.g., age or tumor histology). Thus, the results with respect to both the primary and the secondary objectives should be interpreted with caution.

## Figures and Tables

**Table 1:** Inclusion and exclusion criteria for the APART-LUNG study.

Inclusion criteria	1. Histologically proven lung cancer 2. Treatment with ≥50 Gy of conventionally fractionated radiation therapy 3. Possession of and ability to use a smartphone plus a step counter 4. Age ≥18 years 5. Written informed consent 6. Capacity of the patient to consent
Exclusion criteria	1. Expected non-compliance 2. Cognitive impairment 3. Inability to provide informed consent independently 4. Life expectancy less than the study period

**Table 2:** Overview of enrollment, assessments, and examinations.

	Screening	Baseline	Week 1 of Previous Radiation Therapy	Week 5 of Previous Radiation Therapy
Demographics including age	X			
Parameters of radiation therapy	X			
Medical history and concomitant diseases		X		
Concomitant medication		X	X	
Performance status		X		
Histology of lung cancer		X		
Study-related procedures				
Informed consent	X			
Inclusion criteria	X			
Exclusion criteria	X			
Time the smartphone was worn close to the body at the time of radiation therapy			X	X
Number of steps per day			X	X

## Data Availability

No new data were created or analyzed in this study.

## Abbreviations

The following abbreviations are used in this article:

APART-LUNG	Assessment of Physical Activity during Radiation Therapy for Lung Cancer
CTCAE	Common Terminology Criteria for Adverse Events
GCP	Good Clinical Practice
HeAT	Health Advancing Technologies for the Elderly
ICH	International Council for Harmonisation of Technical Requirements for Pharmaceuticals for Human Use
NSCLC	Non-Small-Cell Lung Cancer
SCLC	Small-Cell Lung Cancer
UKSH	University Medical Center Schleswig-Holstein
